# α-Synuclein serine129 phosphorylation – the physiology of pathology

**DOI:** 10.1186/s13024-023-00680-x

**Published:** 2023-11-13

**Authors:** Nagendran Ramalingam, Ulf Dettmer

**Affiliations:** Ann Romney Center for Neurologic Diseases, Brigham and Women’s Hospital, Harvard Medical School, Boston, MA 02115 USA

**Keywords:** Alpha-synuclein, Phosphorylation, Post-translational modification, Neurotransmission, Synapse, Polo-like kinase-2

## Summary of the message

The study that found phospho-serine129 in the Parkinson’s-linked protein alpha-synuclein over two decades ago proposed a physiological role in regulating protein function, but this notion was neglected when alpha-synuclein serine129 phosphorylation was identified in Lewy bodies/neurites, the hallmark pathology of synucleinopathies. Recent work suggests that both are relevant: pathological phospho-serine129 in Lewy lesions and physiological phospho-serine129 that fine-tunes alpha-synuclein’s synaptic function.

α-Synuclein (αS) phospho-serine129 (pS129) is a specific marker of synucleinopathy – or is it? In the absence of robust antibodies to aggregated αS, it has become a standard to use antibodies to pS129 – in immunohistochemistry, immunocytochemistry, ELISA, or Western blot – and assume that αS aggregation and pS129 are synonyms. Indeed, pathological αS deposits in neurons - Lewy bodies (LBs) and Lewy neurites (LNs) in Parkinson’s disease and Lewy body dementia - were reported to contain about 90% of αS in the pS129 form [1, 3]. However, while there is little doubt that pS129 is present in end-stage aggregates, it has never been clear if pS129 plays any significant role in the cascade of events that lead from native to aggregated αS. On the contrary, recent work suggests pS129 is a late event in pathology, happening to large αS aggregates [4]. In this scenario, pS129 might be irrelevant to the aggregation itself, and could even represent an attempt of the neuron to dissolve the lesions. Consistent with that possibility, it has been proposed that the S129 phosphorylation by Polo-like kinase 2 (Plk2) plays a key role in the degradation of αS [8].

If the notion is true that αS aggregation predates pS129, how should one interpret the widely-documented presence of pS129 under conditions unsuspicious of aggregation: normal human brain, non-human primate brain, and rodent brain [2, 5, 6, 11]? Does αS have such a strong aggregation propensity that even under normal conditions a certain portion misfolds and precipitates? The other (and our preferred) possibility is that pS129 under normal conditions is not pathological at all. Instead, physiological pS129 has evolved to fine-tune αS function, as proposed as early as 2000 [7].

Several lines of evidence, in our view, support the occurrence of physiological pS129 (in addition to the widely documented pathological pS129 associated with αS aggregates). In a recent publication [10], we systematically studied effects of familial PD (fPD) αS missense mutations on basal pS129 in cultured αS knock-out rat neurons. The cultures were transduced with human αS WT, A30P, E46K, H50Q, G51D and A53T. All these mutations, over decades, unequivocally cause PD with classical pathology, including αS S129 hyper-phosphorylation in highly insoluble LB/LN lesions. When expressed for about 2 weeks at roughly physiological levels, however, not even trace amounts of αS were found in highly insoluble biochemical fractions and no uniform pS129 pattern arose: A30P, H50Q, and G51D were all hypo-phosphorylated, E46K αS was hyper-phosphorylated, and A53T was indistinguishable from WT. In the absence of aggregation, pS129 merely correlated with αS-membrane interaction: hypo-phosphorylated A30P, H50Q, and G51D all accumulated in the neurons’ cytosolic fractions. That, as we know, does not make these variants “protective” – in our experimental setup there may just not be enough time for aggregation. In the absence of misfolding, pS129 may be governed by a simple molecular “rule”: membrane-associated αS is a strong, cytosolic αS is a weaker substrate for the kinase (Plk2). Indeed, previous work has shown that pS129 accumulates in membrane fractions, and recombinant αS becomes dramatically more S129-phosphorylated by Plk2 if liposomes are added to the reaction [11].

**Fig. 1:**
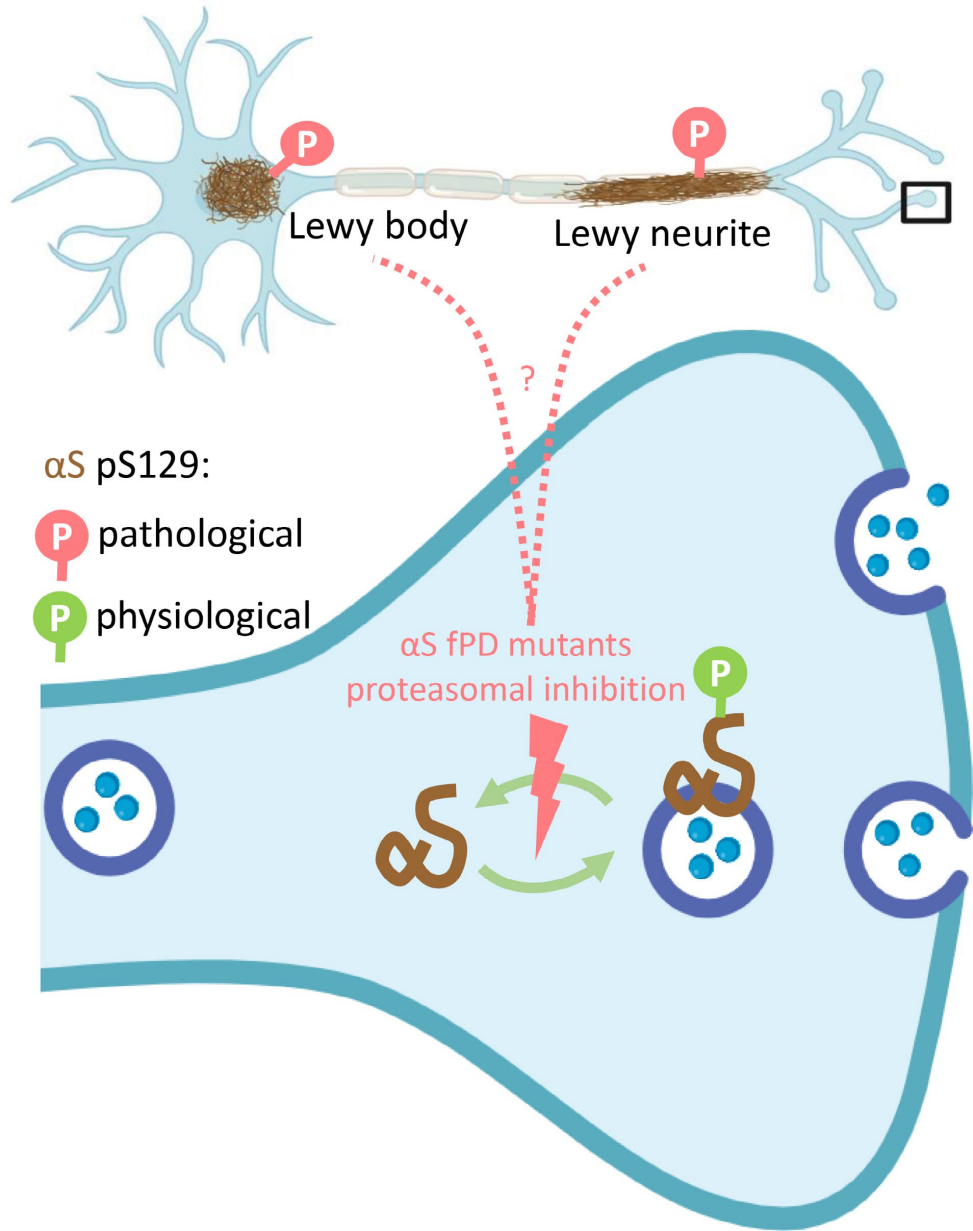
Physiologic vs. pathologic αS serine-129 phosphorylation (pS129). **Top**, Lewy bodies and Lewy neurites are strongly S129-phosphorylated. **Bottom**, dynamic physiological pS129 finetunes synaptic transmission. αS undergoes cycles of phosphorylation and de-phosphorylation as well as membrane attachment and detachment. Membrane-associated αS is preferentially phosphorylated. fPD-linked αS mutations and proteasomal inhibition perturb pS129 dynamics (indicated by thunderbolt). The relationship (if any) between perturbation of pS129 at the synapse and phosphorylation in Lewy lesions requires further research (dotted lines). Created with BioRender.com.

In vivo support for physiological pS129 comes from “enriched-environment” experiments: animals housed under stimulating conditions exhibit both improved long-term potentiation (hippocampal slices) and increased pS129 [11]. It seems difficult to postulate that environmental enrichment causes αS to aggregate.  A more likely explanation is physiological phosphorylation of S129 triggered by neuronal activity. Indeed, stimulating neuron cultures also elevates pS129, readily reversible by inhibiting neuronal activity. So, if physiological pS129 is real, what might be its function? Interestingly, synaptic transmission is impaired in pS129-deficient S129A knock-in mice, consistent with a feed-forward role of pS129. In a study accessible as a preprint, it is suggested that physiological pS129 regulates αS interactions of VAMP2 and synapsin [9], pointing at a possible molecular mechanism. This would be consistent with our finding that activity-dependent pS129 colocalizes with synapsin-containing boutons [11]. If all this is true, do we have to consider αS E46K (hyper-phosphorylated) a stimulator of synaptic transmission and A30P/H50Q/G51D (hypo-phosphorylated) a negative influence? The reality may be more complicated: compared to WT αS, the reversibility of activity-triggered pS129 is reduced for both E46K and A30P, indicating protein dyshomeostasis in both mutants. In addition, proteasomal inhibition also increases pS129, similar to neuronal activity, but with reduced reversibility [10]. More work is needed to establish and understand physiological vs. pathological pS129 (Fig. 1), and it should consider that even in the absence of endpoint aggregation, physiological and pathological pS129 may co-exist. A key question may be: is it all a matter of ratios of principally identical molecular arrangements or is there something unique about non-aggregated, pathological pS129, such as a fold that cannot be considered “normal”?

## Data Availability

N/A.
